# High PEEP Levels during CPR Improve Ventilation without Deleterious Haemodynamic Effects in Pigs

**DOI:** 10.3390/jcm11164921

**Published:** 2022-08-22

**Authors:** Miriam Renz, Leah Müllejans, Julian Riedel, Katja Mohnke, René Rissel, Alexander Ziebart, Bastian Duenges, Erik Kristoffer Hartmann, Robert Ruemmler

**Affiliations:** Department of Anaesthesiology, University Medical Center, Johannes Gutenberg University, Langenbeckstrasse 1, 55131 Mainz, Germany

**Keywords:** resuscitation, porcine, ventilation, MIGET, EIT

## Abstract

Background: Invasive ventilation during cardiopulmonary resuscitation (CPR) is very complex due to unique thoracic pressure conditions. Current guidelines do not provide specific recommendations for ventilation during ongoing chest compressions regarding positive end-expiratory pressure (PEEP). This trial examines the cardiopulmonary effects of PEEP application during CPR. Methods: Forty-two German landrace pigs were anaesthetised, instrumented, and randomised into six intervention groups. Three PEEP levels (0, 8, and 16 mbar) were compared in high standard and ultralow tidal volume ventilation. After the induction of ventricular fibrillation, mechanical chest compressions and ventilation were initiated and maintained for thirty minutes. Blood gases, ventilation/perfusion ratio, and electrical impedance tomography loops were taken repeatedly. Ventilation pressures and haemodynamic parameters were measured continuously. Postmortem lung tissue damage was assessed using the diffuse alveolar damage (DAD) score. Statistical analyses were performed using SPSS, and *p* values <0.05 were considered significant. Results: The driving pressure (P_drive_) showed significantly lower values when using PEEP 16 mbar than when using PEEP 8 mbar (*p* = 0.045) or PEEP 0 mbar (*p* < 0.001) when adjusted for the ventilation mode. Substantially increased overall lung damage was detected in the PEEP 0 mbar group (vs. PEEP 8 mbar, *p* = 0.038; vs. PEEP 16 mbar, *p* = 0.009). No significant differences in mean arterial pressure could be detected. Conclusion: The use of PEEP during CPR seems beneficial because it optimises ventilation pressures and reduces lung damage without significantly compromising blood pressure. Further studies are needed to examine long-term effects in resuscitated animals.

## 1. Introduction

In regard to the use of ventilation in cardiac arrest and cardiopulmonary resuscitation (CPR), specific recommendations regarding optimal respiratory settings remain elusive [1]. Neither the European Resuscitation Council (ERC) nor the American Heart Association (AHA) provide detailed information on ventilation types or the application of positive end-expiratory pressure (PEEP) [2,3]. Guidelines recommend securing the airway during resuscitation and using an endotracheal tube if trained personnel are present. Once the airway is secured, the guidelines suggest using a ventilation rate of 10 breaths per minute and performing continuous chest compressions [2,3]. However, it is known that using PEEP during resuscitation can have positive effects on survival [4] and oxygenation [5]. When using PEEP during CPR, the improved oxygenation is probably due to the prevention of atelectasis [6,7]. In cases with increased extrathoracic pressures, the use of PEEP can redistribute ventilation to the dorsal lung regions [8,9]. However, the continuous application of PEEP could also lead to increased intrathoracic pressures, which can impair venous blood flow [10,11]. However, previous studies showed no impaired venous return when applying continuous PEEP during CPR [4,6,7]. Furthermore, the application of PEEP, in general, aligns with lung-protective ventilation strategies that recommend (among others) the application of a PEEP level greater than 5 cm H_2_O [12], although no sufficient data exist to support the clinical relevance during CPR.

In the presented prospective, randomised, large animal trial, three different PEEP levels were compared using standard (intermittent positive pressure ventilation, IPPV) and low tidal (ultralow tidal volume ventilation, ULTVV) ventilation modes during CPR.

The primary aim of the trial was to examine whether the use of high PEEP levels during CPR can improve gas exchange and optimise ventilation pressures by improving lung recruitment. As a secondary aim, we examined the haemodynamic effects of the applied PEEP levels to determine the clinical value of our findings. Thirdly, we assessed lung tissue damage correlated to the interventions.

## 2. Methods

### Anaesthesia and Instrumentation

This animal trial was approved by the State and Institutional Animal Care Committee Rhineland Palatine (approval no. G20-1-065), and all experiments were performed according to the German Animal Protection Law and the ARRIVE guidelines between January and September 2021. The trial was planned as a prospective, randomised trial.

Forty-two German landrace pigs (age: 12–16 weeks, weight: 29–34 kg) were examined. Sedation, transport, and instrumentation were performed as described in detail before [7]. In short, animals were placed under general anaesthesia using iv injections of fentanyl, propofol, and atracurium, followed by endotracheal intubation. Instrumentation was performed placing iv sheaths in femoral arteries and veins into the left and right groin under sonographic guidance. Additionally, an electrode belt was placed circularly around the thorax, approximately 10 cm above the diaphragm, for electrical impedance tomography measurements (EIT, Pulmo Vista 500, Dräger, Lübeck, Germany).

## 3. Trial Protocol and Data Collection

After induction of anaesthesia and instrumentation, the animals received a fluid bolus of 30 mL/kg balanced electrolyte solution. Six chemically inert gases with different transpulmonary elimination constants (sulphur hexafluoride, krypton, desflurane, enflurane, diethyl ether, acetone) were dissolved in nontoxic doses in saline and given intravenously for the ventilation/perfusion (V/Q) ratio measurements. MIGET was performed after a stabilisation phase of 30 min to reach a steady state.

At the measurement timepoint, baseline healthy (BLH) arterial and central venous blood gases were measured (radiometer, ABL90flex, Copenhagen, Denmark), blood samples for the MIGET measurement (MMIMS-MIGET, Oscillogy LLC, Philadelphia, PA, USA) were taken, and EIT recordings were started. Afterward, the animals received a second dose of atracurium (0.5 mg/kg). The fibrillation catheter was transvenously placed into the right atrium, and continuous ventricular fibrillation was induced with a flicker frequency between 50 and 200 Hertz (Hz). After ECG-confirmed ventricular fibrillation and 5 min of no-flow time, basic life support was started with mechanical chest compressions by the LUCAS 2-System (Stryker, Kalamazoo, MI, USA) with a frequency of 100 compressions/min. Ventilation was performed according to the intervention group. Following the trial protocol, animals were randomised into 6 intervention groups (n = 7 per group, Table 1).

After 30 min of BLS, a rhythm analysis was performed, and guideline-based advanced life support (ALS) was applied if ventricular fibrillation was still detectable. At the CPR measured timepoints of 5 min, 15 min, and 25 min, samples for arterial and central venous blood gas analysis and MIGET measurements were taken. The extended haemodynamic measurements were recorded continuously by using the Datex Ohmeda S5 monitor (GE Healthcare, Munich, Germany). EIT loops were recorded continuously during CPR.

Postmortem lung tissue samples were collected from the cranial, caudal, ventral, and dorsal sections of the left and right lung lobes and fixed with formalin 4%. These samples were paraffinised, cut into 2-micrometre-thick slices, and stained with haematoxylin–eosin (HE) by the tissue bank of the University Medical Center Mainz, Mainz, Germany.

### Scores and Statistics

The histopathologic lung samples were examined with an Olympus microscope (CX43RF, Olympus Cooperation, Tokyo, Japan) via CellSens Software (CellSens Entry.lnk, creation date 3 December 2018) and scored with the previously established diffuse alveolar damage (DAD) score [13]. All statistical planning and interpretations were performed with the assistance of the Institute of Medical Biometrics and Epidemiology of the Johannes Gutenberg University Mainz. Statistical analyses were performed with SPSS (IBM SPSS Statistics, Version: 23 V5 R, Armonk, NY, USA) by using repeated measurements of ANOVA (RMA) and post hoc analysis with Tukey’s test. Statistics of the DAD score were evaluated using linear mixed-effect models. Data in text and graphs are presented as the mean and standard deviation (SD). *p* values lower than 0.05 were considered statistically significant.

## 4. Results

In total, 42 experiments were performed, in which no animal achieved a return of spontaneous circulation (ROSC).

**Figure 1:**
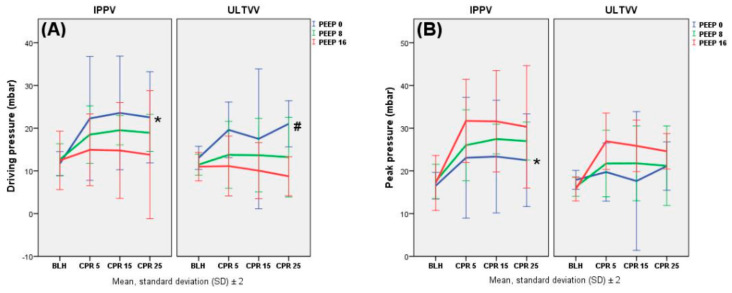
Ventilation: driving pressure (P_drive_, (**A**)), peak pressure (P_peak_, (**B**)). Airway pressures were measured in mbar. Data are shown as mean values and standard deviation (SD). Significant differences in P_drive_ (**A**): * vs. I16, *p* = 0.010; # vs. U16, *p* = 0.003 (Tukey). Significant differences in P_peak_ (**B**) * vs. I16, *p* = 0.011 (Tukey).

The driving pressure (P_drive_) showed a significant difference between the PEEP groups (RMA *p* < 0.001), when adjusting for the ventilation mode: PEEP 16 mbar had significantly lower values than PEEP 8 mbar (Tukey *p* = 0.045) and PEEP 0 mbar (Tukey *p* < 0.001) during CPR. PEEP 8 mbar also showed lower values than PEEP 0 mbar (Tukey *p* = 0.014). The comparison of the six intervention groups showed analogous findings (Tukey I0 vs. I16, *p* = 0.010; Tukey U0 vs. U16, *p* = 0.003). PEEP 16 displayed a marginal mean P_drive_ of 12.23 mbar (± 5.04 mbar), while PEEP 0 mbar showed a marginal mean P_drive_ of 21.06 mbar (± 5.91 mbar). The PEEP groups also showed significant differences when observing the P_mean_ (RMA *p* < 0.001) and P_peak_ (RMA *p* < 0.001) during CPR. PEEP 0 and 8 mbar had significantly lower values than PEEP 16 mbar in P_mean_ (Tukey PEEP 0 mbar vs. PEEP 16 mbar, *p* < 0.001; Tukey PEEP 8 mbar vs. PEEP 16 mbar, *p* < 0.001; Tukey PEEP 0 mbar vs. PEEP 8 mbar, *p* < 0.001) and P_peak_ (Tukey PEEP 0 mbar vs. PEEP 16 mbar, *p* < 0.001; Tukey PEEP 8 mbar vs. PEEP 16 mbar, *p* = 0.031). Similar findings may be observed when comparing the P_mean_ of the intervention groups. In P_peak_, the intervention groups showed a significant difference when comparing I0 vs. I16 (Tukey, *p* = 0.011). PEEP 16 mbar displayed a marginal mean P_peak_ of 28.49 mbar (± 5.28 mbar), while PEEP 0 mbar showed a marginal mean P_peak_ of 21.23 mbar (± 5.89 mbar). Additionally, significant differences were observed when comparing the ventilation modes, adjusted for the PEEP groups, in the P_drive_ (RMA *p* = 0.002), P_mean_ (RMA *p* < 0.001), and P_peak_ (RMA *p* = 0.001) parameters during CPR, with IPPV leading to significantly higher values than ULTVV (Figure 1).

The ventilation–perfusion ratios (V/Q) were measured via MIGET. The U0 group showed non-significantly higher percentages of shunt and significantly higher percentages of low V/Q (Tukey I0 vs. U0, *p* = 0.038) as well as a non-significantly lower fraction of normal and high V/Q during CPR. The I0 group showed non-significantly increasing normal V/Q as well as high V/Q during the intervention and decreased shunt percentage. Both PEEP 16 mbar groups showed decreasing normal V/Q percentages during the intervention and increasing high and low V/Q as well as shunt percentages. The ULTVV groups showed significantly fewer high V/Q (RMA *p* = 0.006) while having significantly more results of low V/Q (RMA *p* = 0.041), adjusted for the PEEP groups.

**Figure 2:**
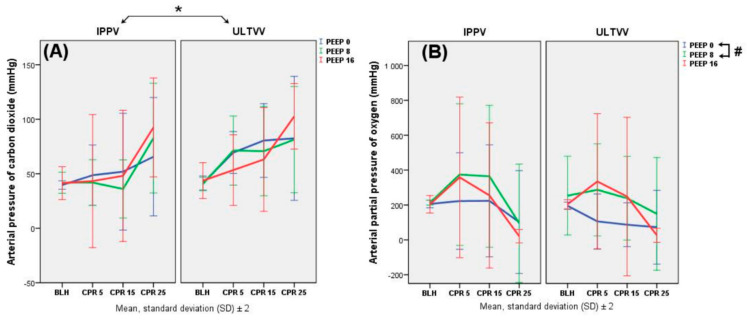
Blood gases: arterial partial pressure of CO_2_ (PaCO_2_, (**A**)), arterial partial pressure of O_2_ (PaO_2_, (**B**)). The unit of PaCO_2_ and PaO_2_ is mmHg. Data are shown as mean values and standard deviation (SD). Significant differences in paCO_2_ (**A**): * ULTVV vs. IPPV, when adjusted for PEEP, *p* = 0.001 (RMA). Significant differences in paO_2_ (**B**): # PEEP 0 mbar vs. PEEP 8 mbar, when adjusted for ventilation mode, *p* = 0.025 (Tukey).

The arterial partial pressure of carbon dioxide (paCO_2_) was significantly higher in the ULTVV mode during the entire intervention (RMA *p* = 0.001), when adjusted for PEEP. At the start of the intervention, the PEEP 0 mbar groups and the U8 group showed high values of paCO_2_, while, at the end, the groups with PEEP 16 mbar displayed the highest paCO_2_ values. The significantly lowest arterial partial pressure of oxygen (paO_2_) was detected in the PEEP 0 mbar group, adjusted for the ventilation mode (Tukey PEEP 0 mbar vs. PEEP 8 mbar, *p* = 0.025). In all intervention groups, the paO_2_ decreased over time (Figure 2).

**Figure 3:**
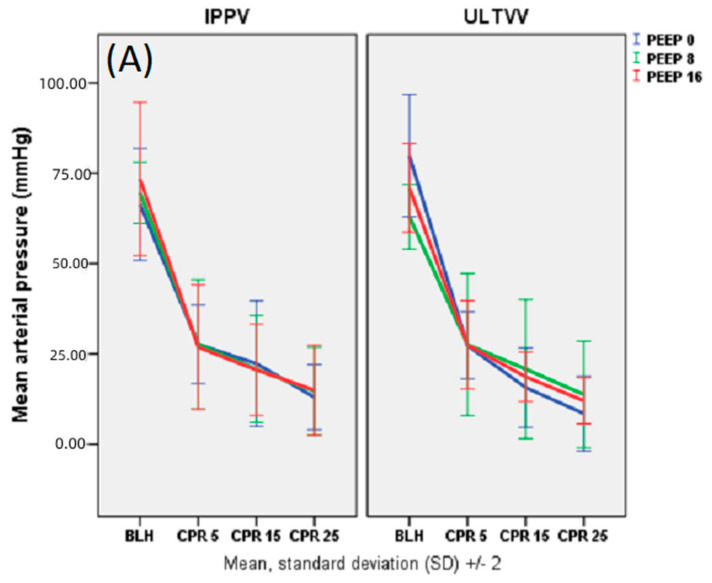
Haemodynamics: mean arterial pressure (MAP, (**A**) The unit of MAP is mmHg. Data are shown as mean values and standard deviation (SD). No significant differences were observed.

There were no significant differences in haemodynamic values between the PEEP groups or tidal volume groups. However, U0 mbar showed a non-significantly lower mean arterial pressure (MAP) during CPR than the groups with PEEP (Figure 3). A detailed summary of cardiopulmonary parameters is shown in Table 2.

The lung physiology was monitored via EIT. In the resulting transverse sectional view, the ROIs are numbered 1 to 4 from the ventral thoracic areas (1) to the dorsal areas (4). During CPR, the highest impedances were observed in ROI 2.

In all ROIs, no significant differences were found between the groups during the intervention. In ROI 1, U0 had non-significantly increased impedances compared with the two ULTTV groups with PEEP. In the dorsal thoracic part, the ULTVV mode displayed non-significantly higher impedances than the IPPV mode when adjusted for the PEEP groups. Here, U0 showed high impedances at the beginning of CPR, which then constantly decreased over time. The highest values in the dorsal thoracic part were observed in the PEEP 16 mbar groups (Supplementary Figure S1).

**Figure 4:**
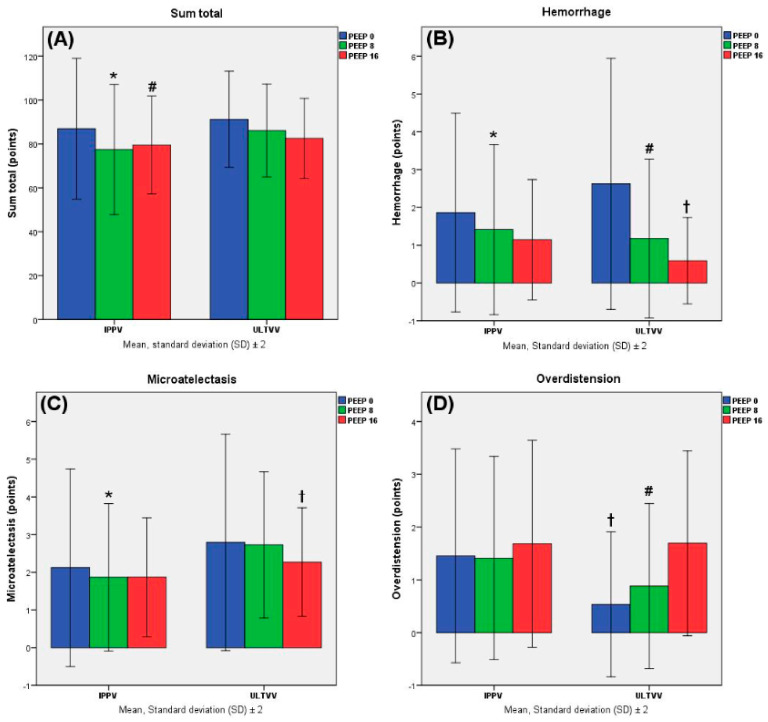
Lung histology evaluated with the DAD score. Categories were scored in points. Data are shown as mean values and standard deviation (SD). Significant differences in sum total (**A**): * vs. I0, *p* = 0.012; # vs. I0, *p* = 0.040 (linear mixed-effect models). Significant differences in haemorrhage (**B**): * vs. I0, *p* = 0.049; # vs. U0, *p* = 0.013; † vs. U0, *p* < 0.001 (linear mixed-effect models). Significant differences in microatelectasis (**C**): * vs. I0, *p* = 0.016; † vs. U0, *p* = 0.013 (linear mixed-effect models). Significant differences in overdistension (**D**): † vs. U16, *p* < 0.001; # vs. U16, *p* = 0.001 (linear mixed-effect models).

Lung histology was evaluated with the DAD score. There was significantly higher lung damage in the sum total category in the PEEP 0 mbar group (all DAD-score-associated significances were evaluated by linear mixed-effect models) (vs. PEEP 8 mbar, *p* = 0.038; vs. PEEP 16 mbar, *p* = 0.009), which could also be observed when comparing the IPPV intervention groups (I0 vs. I8, *p* = 0.012; I0 vs. I16, *p* = 0.040). Nonetheless, the IPPV group showed lower values than the ULTVV group (IPPV vs. ULTVV, *p* = 0.012), which was also observed in an intervention group comparison (I8 vs. U8, *p* = 0.003). Regarding the individual items of the DAD score, the ULTVV mode, adjusted for the PEEP groups, showed greater microatelectrauma (vs. IPPV, *p* < 0.001), while the IPPV group showed more overdistension (vs. ULTVV, *p* = 0.001). In examining the PEEP groups, both PEEP 16 mbar groups showed the greatest overdistension within their ventilation mode (U16 vs. U0, *p* < 0.001; U16 vs. U8, *p* = 0.001). The PEEP 0 mbar groups showed the most microatelectatic tissue in their ventilation mode (I0 vs. I8, *p* = 0.016; U0 vs. U16, *p* = 0.013) as well as haemorrhage (I0 vs. I8, *p* = 0.049; U0 vs. U8, *p* = 0.013, U0 vs. U16, *p* < 0.001) (Figure 4, Supplementary Figure S2).

## 5. Discussion

In this prospective, randomised, controlled animal trial, we examined three different PEEP levels in a standard and low tidal volume ventilation mode during CPR. We discovered that the application of higher PEEP values significantly decreased driving pressures, when adjusted for the ventilation mode. Additionally, we showed that increased PEEP levels did not substantially impair mean arterial blood pressure levels during CPR, suggesting general feasibility while resuscitation efforts via chest compressions are ongoing. EIT and MIGET measurements supported the hypothesis that higher PEEP provides improved recruitment of dependent lung areas, whereas 0 mbar PEEP showed higher histologic damage values with increased atelectrauma.

Ventilation during resuscitation is challenging due to extreme thoracic pressure variances due to chest compressions. The concept of lung-protective ventilation recommends using PEEP greater than 5 cm H_2_O, maintaining P_peak_ below 30 cm H_2_O, and aiming for a low P_drive_, limiting it to 15 cm H_2_O or less [12,14]. In this trial, the intervention groups with PEEP showed significantly lower P_drive_ values during CPR compared to PEEP 0 mbar. When comparing IPPV versus ULTVV, the latter showed significantly lower peak and driving pressures in the respective PEEP groups. This was expected considering that the volume-controlled ventilation mode was set to substantially lower volumes initially. Lower tidal volume strategies lead to less alveolar pressure and thus can avoid overdistention [15]. The reduction in high ventilation pressures can lower the risk of overdistension, which can cause harmful lung damage and may lead to barotrauma [11,12,15].

The histologic examination of both the PEEP 16 mbar groups and all three IPPV groups revealed more overdistended lung tissue, suggesting overinflation and increased stress caused by the increased ventilation pressures necessary to achieve the set tidal volumes. However, the overall lung damage was substantial in the ULTVV mode and the PEEP 0 mbar group, which was mainly driven by significantly higher ratios of bleeding and microatelectasis. This leads to the question of which type of tissue damage—if any—is more crucial for post-ROSC oxygenation and overall outcome. To the best of our knowledge, no concise data are available on this correlation. Using PEEP in general can be beneficial because it leads to better oxygenation and lung recruitment as well as less atelectatic tissue [16]. The paO_2_ values decreased in all six intervention groups during the intervention. However, when using PEEP, increased oxygenation could be observed in this trial. A study that investigated whether different PEEP levels could optimise carbon dioxide clearance during CPR showed that higher PEEP levels lead to significantly decreased paCO_2_ levels and increased minute volume because of a higher fraction of gas oscillations generated by chest compressions [17]. However, this study intentionally did not adhere to resuscitation guidelines regarding respiratory rates, partially explaining these results and potentially reducing their clinical relevance. In our trial, at early resuscitation timepoints, the paCO_2_ was high in the groups without PEEP and the U8 group. The results of the MIGET measurements support the observation of impaired gas exchange when not using PEEP. Especially in the U0 group, overall global hypoventilation with higher shunt perfusion was found. Determinations of high, normal, and low V/Q ratios and shunt volumes were achieved by analysing gas elimination during lung perfusion using a mass spectrometer. The use of MIGET technology during CPR was validated in previous trials [7,18] and can provide additional information about (impaired) circulation and ventilation during CPR. In the IPPV group, a distinct increase in high V/Q volume was observed compared to ULTVV when adjusted for PEEP, which supports an increase in hyperinflated lung areas and is in accordance with previous studies [7]. When using PEEP, lower shunt fractions could be detected, particularly in early resuscitation, suggesting improved recruitment and optimised ventilation. This aligns with studies that showed that using PEEP during resuscitation can prevent airway closure and ensure alveolar ventilation [19,20] and has positive effects on systemic oxygenation [4,5,21]. In our trial, oxygenation was improved when using PEEP, especially in early resuscitation. The ameliorated oxygenation could result from the prevention of atelectasis [4,6,7], thus decreasing shunt perfusion, improving lung recruitment, and preventing airway closure [19,20]. EIT measurements during CPR have not yet been systematically performed and can only yield supportive data, even though general feasibility has been shown previously [22]. Generally, EIT can support the titration of PEEP to avoid atelectasis and prevent regional hyperinflation [23,24,25,26]. In a direct comparison of not using PEEP versus using PEEP, the use of PEEP led to the redistribution of ventilation from the ventral to the dorsal lung regions [8], which could also be suspected in our recordings during CPR, especially in the PEEP 16 groups. Further experimental assessment is needed to validate the method during CPR.

In terms of prolonged CPR, all PEEP groups showed decreasing paO_2_ with an increase in shunt perfusion and lower normal V/Q values. These effects were less pronounced in the PEEP 8 groups, which could emphasise the beneficial effects of moderate PEEP for longer use of CPR.

Interestingly, even when using high PEEP levels, we found no differences in the haemodynamic values, although, theoretically, the continuous application of PEEP and a consecutive high P_peak_ and P_mean_ could lead to increased intrathoracic pressure, which could cause impaired venous return and, consequently, cardiac output (CO) [10,11,21]. A study evaluating the effects of PEEP on CO during CPR suggested a PEEP of 5 mbar as the optimal level in their model [21]. However, the detected decreases—albeit statistically significant—were marginal in absolute values, showing a CO decrease of 0.3 L/min when comparing a PEEP of 0 mbar with 20 mbar, thus determining that the clinical relevance in resuscitation is uncertain. This also aligns with the findings of the present study concerning the MAP, where no significant differences could be detected, which is in line with previous studies focusing on the ULTVV and IPPV modes [7,27], even if only a PEEP of 5 mbar was used in these studies. However, one limitation of our approach is the lack of coronary perfusion pressure measurements (CPP), which is defined as aortic diastolic pressure minus left ventricular end-diastolic pressure and has been linked to positive outcomes and ROSC [28]. Since the primary goal of this study was to evaluate the respiratory effects of higher PEEP during resuscitations, and due to the technical restrictions of our model, these measurements could not be acquired.

In our experiments, no animal achieved ROSC. Since we expanded the uninterrupted chest compression time to 30 min without any drug treatment or defibrillation, this is not surprising, since survival and achievement of ROSC are highly dependent on short intervals leading to first adrenaline dose and shock [29,30].

The use of adrenaline has been shown to increase the rates of survival to hospital admission and long-term survival, although there were no significant differences in cases in which a favourable neurologic outcome was observed [2,31,32]. However, because our trial did not have the specific goal of ROSC, no vasopressors were used during CPR before the 30 min mark, as recommended by the guidelines [2,3]. While there were no differences in MAP when using PEEP, potential effects on survival, neurological outcome, and pulmonary function should be examined in further studies with a focus on shorter resuscitation times, early application of advanced life support algorithms, and post-ROSC monitoring.

## 6. Conclusions

This prospective, randomised, controlled animal trial showed that the use of PEEP during CPR ventilation seems beneficial. It leads to a ventilation pattern with lower driving pressures, optimised ventilation–perfusion ratios, lower shunt perfusion, as well as less atelectatic lung tissue and less overall lung damage. Additionally, no detrimental haemodynamic effects were observed even with high PEEP levels, emphasising the potential benefits of oxygenation without compromising organ perfusion. Further studies are needed to confirm these results and to examine potential long-term effects in resuscitated animals.

## Figures and Tables

**Table 1:** Group design and intervention parameters during resuscitation.

Group Parameters	Group 1–3	Group 4–6
Ventilation mode	IPPV	ULTVV
PEEP level	0, 8, 16 mbar (I0, I8, I16)	0, 8, 16 mbar (U0, U8, U16)
Tidal volume (Vt)	9–10 mL/kgBW	2–3 mL/kgBW
Respiratory rate (RR)	10 breaths/min	50 breaths/min
FiO_2_	1.0	1.0

**Table 2:** Overview of relevant ventilation parameters, invasively measured haemodynamic parameters, blood gases, MIGET measurement, DAD score. Standard deviation (SD), driving pressure (P_drive_), peak pressure (P_peak_), partial arterial pressure of carbon dioxide (paCO_2_), partial arterial pressure of oxygen (paO_2_), mean arterial pressure (MAP), diffuse alveolar damage (DAD) score, overdistension (overdis.), intermittent positive pressure ventilation (IPPV), ultralow tidal volume ventilation (ULTVV). Statistical analyses were performed using SPSS and *p*-values < 0.05 were considered significant. Significant statistical differences are shown with the following symbols: *, #, †.

Parameter	Intervention	CPR 5 min	CPR 15 min	CPR 25 min	*p* Values
	Groups				
MEAN (SD)					
Pdrive	IPPV	18.56 (5.82)	19.27 (6.08)	18.40 (6.35)	
[mbar]	ULTVV *	14.82 (4.96)	13.73 (6.21)	14.31 (6.10)	* vs. IPPV, 0.002
	PEEP 0 mbar	20.93 (5.57)	20.51 (7.82)	21.77 (4.14)	
	PEEP 8 mbar *	16.12 (4.28)	16.58 (4.37)	16.05 (4.57)	* vs. PEEP 0, 0.014
	PEEP 16 mbar ^#,†^	13.02 (4.20)	12.40 (5.04)	11.25 (5.93)	^#^ vs. PEEP 0, 0.000; ^†^ vs. PEEP 8, 0.045
Ppeak	IPPV	26.92 (6.38)	27.46 (6.03)	26.58 (6.03)	
[mbar]	ULTVV *	22.79 (4.57)	21.74 (6.33)	22.29 (3.59)	* vs. IPPV, 0.001
	PEEP 0 mbar	21.39 (5.60)	20.48 (7.70)	21.80 (4.21)	
	PEEP 8 mbar	23.86 (4.45)	24.61 (4.35)	24.07 (4.61)	
	PEEP 16 mbar ^#,†^	29.31 (4.70)	28.71 (5.41)	27.44 (5.87)	^#^ vs. PEEP 0, 0.000; ^†^ vs. PEEP 8, 0.031
shunt	IPPV	19.60 (21.67)	17.42 (14.99)	19.00 (19.85)	
[%]	ULTVV	15.80 (13.56)	17.99 (12.00)	23.06 (11.48)	
	PEEP 0 mbar	29.06 (26.04)	21.59 (12.47)	19.08 (10.87)	
	PEEP 8 mbar	8.78 (4.11)	19.47 (15.43)	14.54 (7.92)	
	PEEP 16 mbar	15.94 (10.95)	12.05 (10.94)	29.47 (22.70)	
paCO_2_	IPPV	44.60 (19.45)	45.32 (24.26)	80.27 (26.36)	
[mmHg]	ULTVV *	64.70 (15.80)	71.43 (20.84)	88.80 (24.27)	* vs. IPPV, 0.001
	PEEP 0 mbar	59.05 (15.75)	66.18 (26.13)	74.02 (28.09)	
	PEEP 8 mbar	56.60 (19.96)	53.35 (24.48)	82.00 (23.79)	
	PEEP 16 mbar	48.30 (24.03)	55.60 (27.22)	97.57 (19.22)	
paO_2_	IPPV	318.38 (197.12)	281.12 (192.31)	72.85 (129.10)	
[mmHg]	ULTVV	242.60 (169.22)	191.30 (163.44)	82.73 (118.34)	
	PEEP 0 mbar	164.26 (123.97)	155.42 (136.91)	87.27 (124.16)	
	PEEP 8 mbar ^#^	330.57 (170.61)	301.68 (173.26)	122.38 (161.54)	^#^ vs. PEEP 0, 0.025
	PEEP 16 mbar	346.63 (205.03)	251.53 (209.41)	23.71 (19.15)	
MAP	IPPV	27.38 (7.42)	21.22 (7.17)	14.19 (5.45)	
[mmHg]	ULTVV	27.46 (6.82)	18.38 (6.70)	11.43 (5.69)	
	PEEP 0 mbar	27.48 (4.84)	18.99 (7.77)	10.73 (5.21)	
	PEEP 8 mbar	27.62 (9.02)	20.79 (8.22)	14.20 (6.51)	
	PEEP 16 mbar	27.17 (7.18)	19.62 (4.98)	13.49 (5.01)	
		**Post mortem**			
DAD.	IPPV	1.95 (1.04)			
atelectasis	ULTVV *	2.59 (1.10)			* vs. IPPV, 0.000
[points]	PEEP 0 mbar	2.45 (1.40)			
	PEEP 8 mbar ^#^	2.29 (1.06)			^#^ vs. PEEP 0, 0.047
	PEEP 16 mbar	2.06 (0.77)			
DAD.	IPPV	1.51 (0.98)			
overdistens.	ULTVV *	1.03 (0.91)			^#^ vs. IPPV, 0.001
[points]	PEEP 0 mbar	0.99 (0.97)			
	PEEP 8 mbar	1.14 (0.91)			
	PEEP 16 mbar ^#,†^	1.68 (0.92)			^#^ vs. PEEP 0, 0.000; ^†^ vs. PEEP 8, 0.012

## Data Availability

All data analysed for this study are provided in the manuscript and the Supplementary Materials.

## Supplementary Materials

The following supporting information can be downloaded at: https://www.mdpi.com/article/10.3390/jcm11164921/s1, Figure S1: Lung physiology via Electrical impedance tomography (EIT): the picture shows EIT loops during CPR at the intervention timepoint CPR 5 min. A = ULTVV PEEP 0 mbar, B = IPPV PEEP 0 mbar, C = ULTVV PEEP 16 mbar, D = IPPV PEEP 16 mbar. Global EIT recordings and recordings of ROI (region of interest) 1 to ROI 4 are pictured. There were no significant differences detected. Figure S2: Microphotographs of histologic lung samples with the 7 aspects of the DAD-Score used.
