# Supplementary material for: High PEEP Levels during CPR Improve Ventilation without Deleterious Haemodynamic Effects in Pigs
Source: J Clin Med. 2022 Aug 22;11(16):4921. doi: 10.3390/jcm11164921 (PMC9410261; doi:10.3390/jcm11164921)
Supplement: Supplementary file 1 [file jcm-11-04921-s001.zip › jcm-1841250-supplementary.pdf]

Supplemental data to

High PEEP Levels during CPR Improve Ventilation without Deleterious Hemodynamic Effects in Pigs

Miriam Renz[1], Leah Müllejans[1], Julian Riedel[1], Katja Mohnke[1], René Rissel[1], Alexander Ziebart[1], Erik Kristoffer Hartmann[1] and Robert Ruemmler[1]

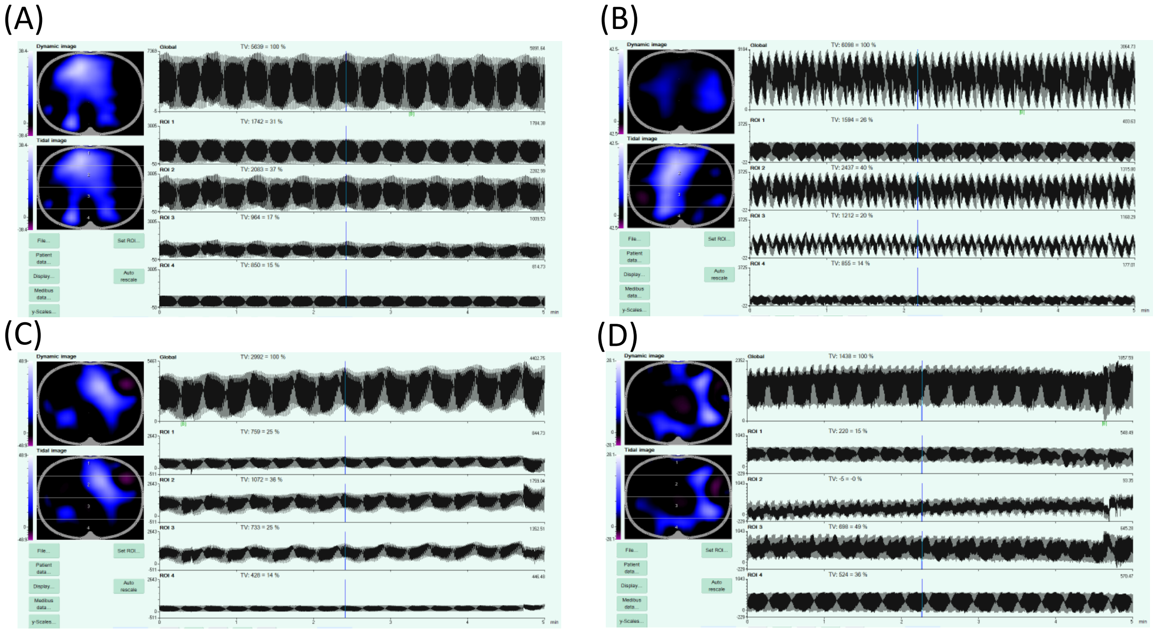

Figure S1. Lung physiology via Electrical impedance tomography (EIT): the picture shows EIT loops during CPR at the intervention timepoint CPR 5 min. A = ULTVV PEEP 0 mbar, B = IPPV PEEP 0 mbar, C = ULTVV PEEP 16 mbar, D = IPPV PEEP 16 mbar. Global EIT recordings and recordings of ROI (region of interest) 1 to ROI 4 are pictured. There were no significant differences detected.

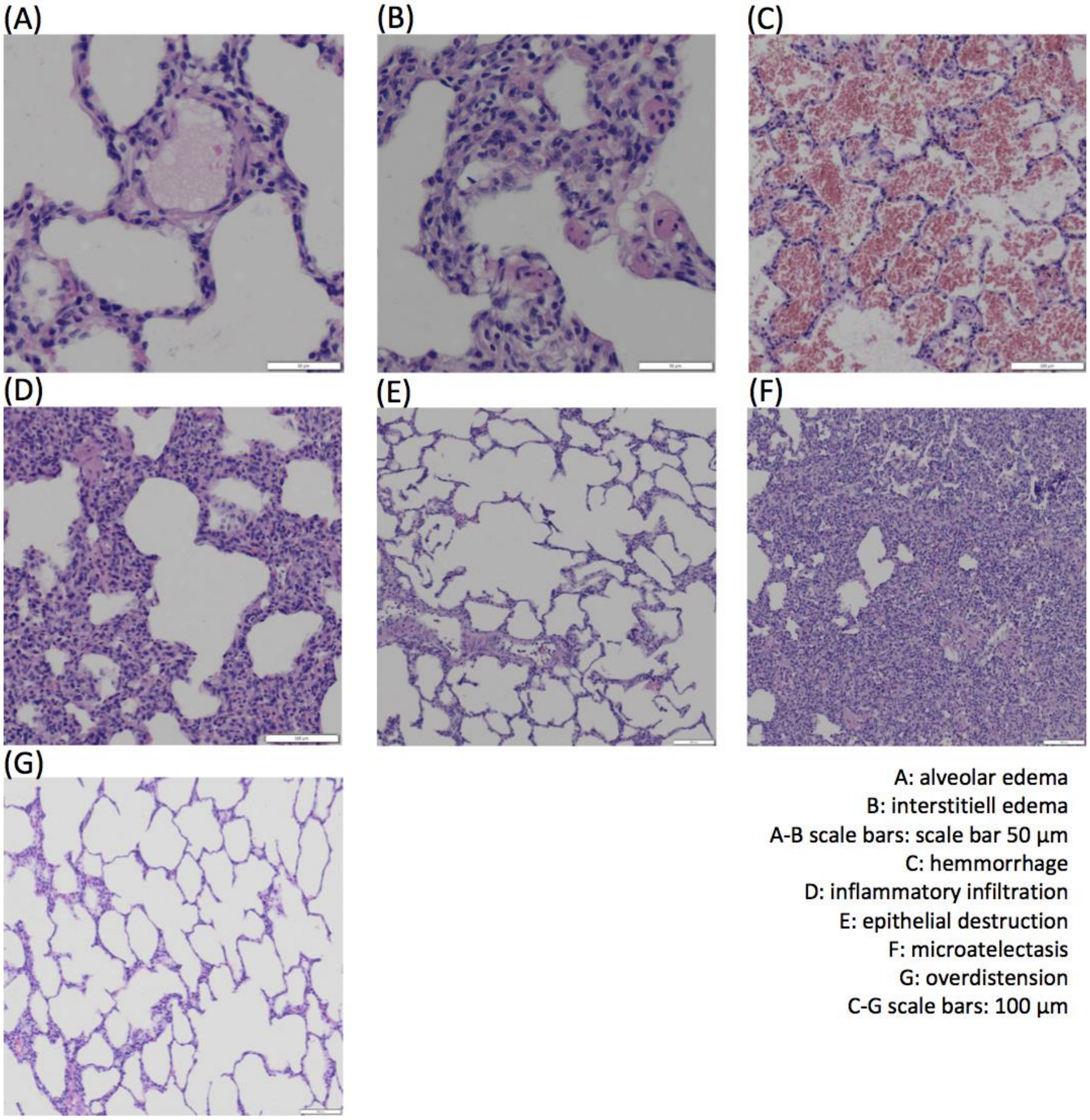

Figure S2. Microphotographs of histologic lung samples with the 7 aspects of the DAD-Score used.
